# *Candida auris:* estrategias y retos para prevenir un brote

**Published:** 2020-03-30

**Authors:** Alejandra Zuluaga-Rodríguez

**Affiliations:** 1 Unidad de Micología Médica y Experimental, Corporación para Investigaciones Biológicas (CIB), Medellín, Colombia azuluaga@cib.org.co Corporación para Investiga. Biológicas Corporación para Investigaciones Biológicas Medellín Colombia azuluaga@cib.org.co

*Candida auris* es una levadura emergente multirresistente que se ha asociado a brotes de infecciones intrahospitalarias alrededor del mundo ^1^, incluyendo instituciones de salud de Colombia ^2^^-^^4^. El Instituto Nacional de Salud reportó, entre febrero de 2015 y mayo de 2017, 123 casos de infecciones por *C. auris* en Colombia. Estos casos se identificaron en 24 centros hospitalarios de nueve departamentos del país y el 60 % de ellos se asociaron con infecciones del torrente sanguíneo. Al momento de dicho reporte, la mitad de los casos habían sido identificados en instituciones médicas de la Costa Caribe del país (Atlántico, Bolívar y Cesar) ^5^.

Los factores de riesgo asociados al desarrollo de infecciones invasivas por *C. auris,* son similares a los asociados a infecciones por otras especies del género *Candida,* los cuales incluyen: procedimientos quirúrgicos mayores, antibióticos de amplio espectro, hospitalizaciones prolongadas, presencia de dispositivos invasivos, como tubos para asistencia respiratoria mecánica y para alimentación, y catéteres venosos centrales. Además, es bien conocido que la mayoría de las infecciones invasivas por especies de este género, están relacionadas con pacientes en estado crítico, es decir, en unidades de cuidados intensivos y sometidos a procedimientos invasivos, o pacientes con condiciones médicas de base, como diabetes, neoplasias hematológicas y otras inmunosupresiones ^6^^,^^7^.

Los retos para el control de *C. auris* podrían resumirse en tres componentes principales, a saber:

1) la identificación incorrecta del microorganismo por parte del laboratorio,

2) la multirresistencia a los antifúngicos, y

3) un hongo con capacidad de generar brotes intrahospitalarios.

## Identificación incorrecta del microorganismo por parte del laboratorio

La capacidad de identificar con precisión a *C. auris* continúa siendo un desafío, ya que la mayoría de los métodos comerciales basados en identificación fenotípica, como Vitek 2, BD Phoenix, API 20C AUX y MicroScan, identifican de forma errónea los aislamientos de *C. auris,* especialmente *C. haemulonii, C. sake, Rhodotorula glutinis* u otras especies de *Candida* spp. ^8^. Recientemente, Vitek 2 ha actualizado su base de datos (versión 8.01), la cual incluye a *C. auris.* Sin embargo, es importante tener en cuenta que esta nueva base de datos, si bien es capaz de identificar correctamente los aislamientos de *C. auris* pertenecientes al clado suramericano, aún sigue presentando identificaciones erróneas para los aislamientos pertenecientes a otros clados ^9^^,^^10^.

Por el momento, la identificación más precisa de *C. auris* se puede lograr utilizando: i) MALDI-TOF MS, ii) PCR específicas para *C. auris,* iii) secuenciación de la región D1/D2 de la subunidad de ARN ribosómico del complejo de genes *28S,* o iv) regiones espaciadoras transcritas internas del ADN ribosómico ^11^^-^^14^, herramientas con tiempos de respuesta rápidos, pero cuyo costo y la habilidad involucrados en su adquisición y operación siguen siendo un obstáculo para la mayoría de los laboratorios de micología ^15^^,^^16^, lo cual hace necesario remitir los aislamientos sospechosos a los centros de referencia con disponibilidad de dichas herramientas y, de esta forma, corroborar las identificaciones.

## Multirresistencia a los antifúngicos

A pesar de no existir puntos de corte aprobados por el *Clinical and Laboratory Standards Institute* (CLSI) y la *European Committee for Antimicrobial Susceptibility Testing* (EUCAST), se ha observado que *C. auris* presenta una sensibilidad reducida a los antifúngicos. Dada la emergencia global, los *Centers for Disease Control and Prevention* (CDC) han propuesto de forma temporal utilizar las siguientes concentraciones inhibitorias mínimas (CIM) como puntos de corte: ≥32 ug/ml para el fluconazol, ≥2 µg/ml para la anfotericina B (o ≥1,5 utilizando Etest ug/ml), ≥4 ug/ml para la anidulafungina y la micafungina, y ≥2 ug/ml para la caspofungina ^17^.

## Hongo con capacidad de generar brotes intrahospitalarios

La capacidad de colonizar la piel de pacientes y sus contactos, y la capacidad de contaminar el ambiente y persistir en él, son dos aspectos clave de *C. auris* para generar brotes en los centros de salud. La colonización con *C. auris* se ha detectado en múltiples sitios del cuerpo: fosas nasales, ingles, axilas y recto. En algunos reportes se han descrito aislamientos de *C. auris* durante tres meses o más después de su detección inicial ^6^^,^^18^^,^^19^. Los factores de riesgo para la colonización incluyen el contacto con otros pacientes colonizados por *C. auris* y con un ambiente hospitalario contaminado por este microorganismo ^20^.

Dado lo anterior, se evidencia la importancia de implementar medidas de control para frenar la transmisión y controlar los brotes de *C. auris* en las instituciones de salud; cualquier retraso en la implementación de dichas medidas o actividades de control de infecciones, puede provocar una transmisión rápida y constante de *C. auris* entre los pacientes ^8^.

La implementación de estas prácticas comienza con la identificación de los casos. Este microorganismo es transmisible, tanto en pacientes enfermos como en los colonizados asintomáticos; por lo tanto, las precauciones de control de la infección son las mismas para todos los pacientes. La identificación de *C. auris* en cualquier sitio del cuerpo puede representar una fuente de transmisión y debe generar la implementación de las medidas de control de infecciones en el centro médico donde se encuentre el paciente ^9^. La detección se hace comúnmente con hisopados tomados de las axilas y las ingles, ya que se ha determinado que en estos sitios anatómicos se aísla con mayor frecuencia ^21^. Otros sitios del cuerpo de los cuales se ha aislado *C. auris* son la nariz, la boca y los conductos auditivos externos, y, asimismo, se ha hecho de la orina, las heridas y el recto.

Los CDC han publicado varias recomendaciones para el control de las infecciones por *C. auris* que incluyen identificación de casos, higiene de manos, precauciones basadas en la transmisión y limpieza ambiental ^8^^,^^9^^,^^21^.

### 1. Identificación de casos

- Identificar las especies de *Candida* aisladas de sitios estériles.
- Identificar las especies de *Candida* aisladas de sitios no estériles cuando el paciente se encuentre en una institución de salud donde se han identificado casos de *C. auris,* o provenga de una institución donde se hayan detectado casos, tanto en el país de origen como en el extranjero, especialmente si es un país donde se han reportado casos de transmisión de *C. auris.*

Se debe considerar el examen de pacientes que:

- Son contactos cercanos de los casos.
- Han recibido atención médica en el extranjero, especialmente en un país con casos de *C. auris.*
- Si se sospecha la transmisión, el centro de atención médica debe considerar la posibilidad de ampliar la detección incluyendo todas las personas de la sala en donde se hayan identificado los casos.
- Las intervenciones de control de infecciones son las mismas para pacientes con infección por *C. auris* y para aquellos asintomáticos con colonización.

### 2. Higiene de manos

- El personal de salud debe practicar una higiene de manos adecuada y frecuente.
- El uso de desinfectantes a base de alcohol es eficaz contra *C. auris* y se recomienda para la higiene de las manos de forma rutinaria. En caso de que estén visiblemente sucias, se recomienda lavarse las manos con agua y jabón.
- Supervisar la observancia de las prácticas de higiene de manos por parte del personal de salud.

### 3. Precauciones basadas en la transmisión

- En caso de ser posible, se deben hospitalizar a los pacientes con *C. auris* en habitaciones individuales. Si hay un número limitado de habitaciones individuales, estas se deben reservar para aquellos con mayor riesgo de transmitir *C. auris,* especialmente, los que requieren más atención.
- Los pacientes con *C. auris* pueden ser hospitalizados en habitaciones con otros que también se encuentren infectados con *C. auris,* pero no se recomienda ubicar los infectados con *C. auris* con pacientes infectados con otros agentes.
- En lo posible, se debe minimizar el número de personas que atienden a los pacientes con *C. auris.* Si hay varios pacientes con esta infección en un establecimiento, se debe considerar reducir el número de miembros del personal que los cuidan a un grupo limitado.
- Supervisar la observancia de las precauciones basadas en la transmisión por parte del personal de salud.

## 4. Limpieza ambiental

En varios estudios se ha demostrado la persistencia de *C. auris* en el ambiente, por lo tanto, la correcta limpieza es clave para el control de los brotes ^17^^-^^19^.

Al momento de llevar a cabo las actividades de limpieza ambiental, se recomienda tener en cuenta lo siguiente:

- Los productos que contengan amonios cuaternarios, usados comúnmente para la desinfección, no son eficaces contra *C. auris.*
- Se recomienda usar desinfectantes de grado hospitalario que estén registrados en la *Environmental Protection Agency* (EPA) de los Estados Unidos y que sean eficaces contra las esporas de *Clostridium difficile*^22^*.* Recientemente, la EPA ha aprobado aprobó los siguientes productos:

■ Oxivir 1, limpiador a base de peróxido de hidrógeno; presentación en solución (registro EPA 70627-74) y en toallas limpiadoras (registro 70627-77).

■ Micro-Kill, producto a base de hipoclorito de sodio; presentación en toallas germicidas (registro EPA 37549-1) ^22^^,^^23^.

- Otros productos recomendados por los CDC y la EPA, pero que aún no tienen un registro específico para *C. auris,* se encuentran resumidos en: https://www.cdc.gov/fungal/candida-auris/pdf/section-18-public-health-exemption-508.pdf. Los principales ingredientes activos de estos productos son peróxido de hidrógeno, hipoclorito de sodio, combinaciones de peróxido de hidrógeno con ácido peroxiacético, y combinaciones de amonios cuaternarios con etanol e isopropanol.
- Es muy importante seguir todas las instrucciones del fabricante sobre el uso de los desinfectantes, incluidos la preparación y conservación del producto y el tiempo de contacto.
- Se debe realizar limpieza y desinfección minuciosa diariamente en las áreas de atención a los pacientes con *C. auris.*
- Es necesario hacer la limpieza y desinfección de las áreas externas a la habitación en donde los pacientes reciben atención médica, por ejemplo, las salas de cirugía y las de rayos X, entre otras.
- Se deben limpiar y desinfectar los aparatos que se comparten, entre ellos, los respiradores, los termómetros, los tensiómetros y los equipos de fisioterapia. Esto se debe hacer antes y después de cada uso.

No existe información concluyente sobre la efectividad de los protocolos para la descolonización de los pacientes con *C. auris.* La clorhexidina al 2 % en toallas desechables o al 4% en solución acuosa, fue utilizada con buenos resultados durante un brote en el Reino Unido ^18^^,^^21^. En los pacientes con asistencia respiratoria mecánica, la clorhexidina en enjuague bucal al 0,2 %, en gel dental al 1 % o para administración oral, también ha demostrado buenos resultados. Igualmente, se pueden utilizar discos impregnados con clorhexidina al momento insertar catéteres venosos centrales ^18^^,^^21^.

Por el momento, el tratamiento de *C. auris* es el mismo recomendado para las infecciones causadas por otras especies del género *Candida.* Para más detalles, se recomienda consultar las guías de la *Infectious Diseases Society of America* (IDSA), publicadas en el 2016 ^24^. Asimismo, es de gran importancia que las infecciones por *C. auris* sean manejadas por un especialista en enfermedades infecciosas.

## Conclusión

Considerando lo anterior y teniendo en cuenta que los casos por *C. auris* continúan notificándose alrededor del mundo y en instituciones de salud en Colombia, se hace necesario fortalecer la capacidad de sospecha y detección de este microorganismo. Asimismo, es importante resaltar que la correcta identificación de esta levadura es el punto de partida para la detección y el control de los brotes, lo cual también implica un manejo adecuado del paciente. Todo esto se requiere para controlar su transmisión en el ambiente hospitalario y, finalmente, lograr reducir la actual y preocupante propagación hospitalaria que se viene observando.
